# Tumor matrix remodeling and novel immunotherapies: the promise of matrix-derived immune biomarkers

**DOI:** 10.1186/s40425-018-0376-0

**Published:** 2018-07-03

**Authors:** Muhammad Umair Mushtaq, Athanasios Papadas, Adam Pagenkopf, Evan Flietner, Zachary Morrow, Sibgha Gull Chaudhary, Fotis Asimakopoulos

**Affiliations:** 1 Department of Medicine, University of Wisconsin School of Medicine and Public Health, Madison, WI USA; 2 University of Wisconsin Carbone Cancer Center, 1111 Highland Avenue, WIMR 4031, Madison, WI 53705 USA

**Keywords:** Adaptive immune response, Immune checkpoint inhibitors, Immunotherapy, Tumor microenvironment, Matrix remodeling, Immune biomarkers

## Abstract

Recent advances in our understanding of the dynamics of cellular cross-talk have highlighted the significance of host-versus-tumor effect that can be harnessed with immune therapies. Tumors exploit immune checkpoints to evade adaptive immune responses. Cancer immunotherapy has witnessed a revolution in the past decade with the development of immune checkpoint inhibitors (ICIs), monoclonal antibodies against cytotoxic T lymphocyte antigen 4 (CTLA-4) and programmed cell death protein 1 (PD-1) or their ligands, such as PD1 ligand 1 (PD-L1). ICIs have been reported to have activity against a broad range of tumor types, in both solid organ and hematologic malignancy contexts. However, less than one-third of the patients achieve a durable and meaningful treatment response. Expression of immune checkpoint ligands (e.g., PD-L1), mutational burden and tumor-infiltrating lymphocytes are currently used as biomarkers for predicting response to ICIs. However, they do not reliably predict which patients will benefit from these therapies. There is dire need to discover novel biomarkers to predict treatment efficacy and to identify areas for development of combination strategies to improve response rates. Emerging evidence suggests key roles of tumor extracellular matrix (ECM) components and their proteolytic remodeling products in regulating each step of the cancer-immunity cycle. Here we review tumor matrix dynamics and matrix remodeling in context of anti-tumor immune responses and immunotherapy and propose the exploration of matrix-based biomarkers to identify candidates for immune therapy.

## Background

The adaptive immune response protects against foreign threats, including infections and tumors. The therapeutic potential of host-versus-tumor effect can be harnessed with novel immune therapies. CD4^+^ and CD8^+^ T lymphocytes comprise primary effector cells against tumors. Initial antigen-mediated activation of T cells is modulated by several regulatory mechanisms, including engagement co-stimulatory signals like the binding of CD28 on T cells to CD80/B7-1 and/or CD86/B7-2 on antigen-presenting cells. Immune checkpoint pathways that have evolved as a mechanism to avoid auto-immunity, can be targeted with immune checkpoint inhibitors (ICIs). Immune checkpoints are inhibitory regulators that act as “breaks” on the immune response. Cytotoxic T lymphocyte antigen 4 (CTLA-4; CD152) competes with CD28 for the ligands CD80 and CD86, and antagonizes T cell receptor (TCR) signaling [1–3]. Programmed cell death protein 1 (PD-1; CD279) counters positive signaling by the TCR by engaging its ligands programmed cell death 1 ligand 1 (PD-L1; CD274/B7-H1), and/or PD-L2 (CD273/B7-DC) [4–7]. The generation of an inflammatory milieu in the tumor microenvironment (TME) and infiltration of activated lymphocytes induce tumor escape mechanisms that exploit immune checkpoints to evade adaptive immune responses, including up-regulation of PD-L1 in TME and CTLA-4 in peripheral lymphoid tissues [8–10].

### Immune checkpoint inhibitors: urgent need for predictive biomarkers

Tumor immunotherapy has witnessed a revolution in the past decade. The clinical successes of ICIs, monoclonal antibodies (mAb) against CTLA-4 and PD-1 pathways, was a breakthrough achievement. In 2010, a randomized phase III trial reported remarkable response to Ipilimumab, mAb against CTLA-4, in melanoma patients [11]. Ipilimumab was the first checkpoint inhibitor to be approved by the Food and Drug Administration (FDA). Pembrolizumab and nivolumab, mAbs against PD-1, were FDA-approved in 2014. Atezolizumab, mAb against PD-L1, was FDA-approved in 2016. Two mAbs to PD-L1, Durvalumab and Avelumab, were granted breakthrough FDA approval in 2017 after promising results in non-small cell lung cancer (NSCLC), urothelial carcinoma and Merkel cell carcinoma [12–14].

Despite the rapid progress of approvals for these classes of agents, the accumulated experience demonstrated that overall, only one-third of the patients achieve a durable and meaningful response. With CTLA-4 blockade by Ipilimumab or PD-1 inhibition with Nivolumab, response rates of 30-40% were observed in melanoma patients as monotherapies and combination therapy achieved a response rate of over 50% [15–18]. In NSCLC, a response rate of about 20% is observed with Nivolumab, Pembrolizumab and Atezolizumab [19–22]. Response rates of 13% (head and neck squamous cell cancer), 25%-40% (renal cell cancer), and 31% (microsatellite-unstable colon cancers) have been reported with PD-1 blockade [23–25]. In relapsed/refractory Hodgkin's lymphoma, a complete response rate of 17% and partial response rate of 70% has been reported with Nivolumab [26]. A complete remission rate of 22% is noted in relapsed/refractory acute myeloid leukemia with Nivolumab combined with a hypomethylating agent [27]. There are several on-going bench and clinical trials for ICIs across all tumor types. However, it is clear that to-date, the majority of patients do not benefit from checkpoint inhibition immunotherapy. There is dire need to explore biomarkers to predict response to treatment and to identify areas for development of combination agents to improve response rates and mitigate toxicities.

### Predictors of response to immune checkpoint inhibitors: current limitations

#### Expression of immune checkpoints: challenges and pitfalls

High expression of PD-L1 is regarded as a marker of an active anti-tumor immune response and correlates with adaptive immune resistance in several tumor types, including melanoma, NSCLC, Merkel cell carcinoma, breast cancer, mismatch-repair deficient tumors, and Hodgkin's lymphoma [10, 19, 21, 22, 26, 28–34]. However, expression of PD-L1 does not reliably predict response to ICI [18, 35, 36]. In NSCLC, no association of PD-L1 expression with response has been reported with Nivolumab [20]; however, high PD-L1 expression in NSCLC almost doubled the response rate to Pembrolizumab to about 45% from 19% [37]. In melanoma, tumor tissue PD-L1 expression showed significant correlation with response in five out of eight PD-1 ICI studies but did not predict response to CTLA-4 ICI therapy [38]. Notably, there are several limitations concerning PD-L1 expression assays, including membranous versus cytoplasmic expression, expression by multiple cell types in the TME, focal expression in tumor samples, changes in expression over the course of disease progression and with radiation and epigenetic chemotherapy, as well as variability in laboratory techniques and antibodies used in the assay [35].

Discordance between PD-L1 expression in metastatic sites and primary tumors has been noted in bladder cancer patients, suggesting the dynamic nature of TME [39]. In contrast to pre-treatment biopsies, tumor biopsies in early treatment phase in metastatic melanoma patients treated with sequential CTLA-4 and PD-1 blockade showed high expression of PD-1 and PD-L1 in responders [40]. In NSCLC cells, PD-L1 genomic locus amplification correlated with expression of PD-L1 and antitumor benefit [41]. CTLA-4 and PD-L2 genes were expressed at higher levels in the pretreatment melanoma tumors of patients who derived benefit from CTLA-4 antibodies [42]. However, PD-L1, PD-L2 and CTLA-4 did not demonstrate higher expression in anti-PD-1-responsive melanoma patients [43].

#### Somatic mutations and neoantigen load

A systemic review of melanoma patients showed that responses to ICIs correlated with mutational load, neoantigen load, and immune-related gene expression [38]. High mutational burden and neo-epitope density have been noted in responding tumors; however, there is significant overlap with non-responding tumors [34, 42, 44]. Colon cancers with microsatellite instability (MSI) have large mutational burdens and higher response rates to PD-1 blockade [23, 33]. However, high mutational burdens do not always predict responders to ICI therapy, primarily because of an extremely diverse array of resultant somatic mutations [34, 42–44]. Neoantigen heterogeneity influences immune surveillance. Clonal neoantigens have been reported to induce immune reactivity and sensitivity to immune checkpoint blockade [45].

#### Immune profiling signatures

Genetic and immune heterogeneity has been observed in melanoma tumors responding to immunotherapy [46]: individual gene-based expression analysis has revealed that mesenchymal and T cell-suppressive inflammatory or angiogenic tumor phenotypes are associated with innate anti-PD-1 resistance [43]. Genes expressed higher in non-responding pre-treatment tumors included mesenchymal transition genes, immunosuppressive genes, and monocyte and macrophage chemotactic genes [43].

#### Tumor-infiltrating cytotoxic lymphocytes (CTL)

The success of checkpoint blockade depends on prior recruitment of tumor-infiltrating lymphocytes, particularly CD8^+^ cytotoxic T- lymphocytes (CTL), in the TME. These CTL are located at the invasive tumor margin and intratumorally, and are negatively regulated by PD-1/PD-L1-mediated adaptive immune resistance. In metastatic melanoma, the detection of CTL at the tumor margin predicted better response to ICI [10, 38, 40, 47]. Colon cancers with MSI are highly infiltrated with T cells relative to microsatellite-stable (MSS) colon cancers, particularly with CTL [48]. Chemokines of CCL and CXCL family have been associated with CTL recruitment to melanoma metastases [49]. Higher levels of CCL2, CXCL4 and CXCL12 have been noted in responding tumors [47]. Clonal T cell responses have been associated with ICI clinical responses [10, 50–52]. It remains unclear how ICIs affect CD8^+^ effector memory cells that might explain durable response observed in many patients [53]. Conversely, brisk CTL infiltrates at time of progression in patients on immune checkpoint blockade has also been noted, suggesting that effector immune cells are impaired by the TME leading to therapeutic resistance [54].

#### Tumor-infiltrating regulatory T cells (Tregs)

Tumor-infiltrating Tregs, in particular, CD4^+^ T cells expressing interleukin-2 receptor chair-alpha (IL2Rα; CD25) and transcription factor forkhead-box P3 (FOXP3), suppress CTL and contribute to a tumorigenic TME. They promote tumor growth by diverse mechanisms, including expression of immune checkpoints (CTLA-4, PD-1 and others) as well as production of IL10 and transforming growth factor-beta (TGF-β) [55, 56]. CTLA-4 blockade expands the population of Tregs and high levels of soluble CD25 (IL2Rα) has been correlated with resistance to anti-CTLA-4 therapy [57]. PD-1 blockade with Nivolumab promoted CTL proliferation and resistance to Treg-mediated suppression, by down-regulating intracellular expression of FOXP3 [58]. An increased ratio of CTL compared with Treg in tumor tissue has been associated with response to CTLA-4 and PD-1 blockade [27, 59].

#### Tumor-infiltrating regulatory myeloid cells

Tumor-infiltrating myeloid cells, including myeloid-derived-suppressor cells (MDSCs), tumor-associated granulocytes, tumor-associated macrophages (TAMs) and dendritic cells (DCs), generate and promote both immunogenic and tolerogenic responses [60–63]. MDSCs are immune-suppressive immature myeloid cells that support tumor growth and predict poor prognosis [64–67]. MDSCs exert their effects by various mechanisms including arginine 1 expression [68], nitric oxide [69], cyclooxygenase 2 [70], reactive oxygen species [71], and Treg activation via CD40–CD40L interactions [72]. In melanoma, an elevated level of CXCL17, which recruits MDSCs, predicts non-responders to ICI [47, 73].

Tumor-associated neutrophils (TANs) and TAMs have been classified to have an anti-tumor (type 1) or pro-tumor (type 2) phenotype [74–77]. Pro-tumor effects of TANs include dampening of CTL response, increased angiogenesis, and modulation of cellular trafficking [78]. Type 1 TAMs (M1) produce immune-stimulatory cytokines, like IL6, IL12 and CXCL9, that promote infiltration of CTLs whereas type 2 TAMs (M2) support tumor growth by diverse pathways, including production of angiogenic factors like IL-10 and CCL22, matrix remodeling by proteases, and inhibition of CTLs and DCs [79]. PD-L1 expression by monocytes and TAMs promote immune evasion and correlate with disease progression in hepatocellular carcinoma [80]. Fc-gamma receptors (FcγRs) expressed by M2 TAMs facilitate anti-tumor response to CTLA-4 inhibition through Treg depletion [81, 82]. Tumor-infiltrating eosinophils promote infiltration of CTLs, by varied machnisms, including polarization of TAMs and normalization of tumor vasculature, and predict a better prognosis in colon cancer [83, 84]. Tumor-infiltrating mast cells recruit MDSCs and upregulate production of pro-inflammatory cytokines resulting in Treg infiltration and immune suppression [85–87].

DCs, including classical (cDCs) and plasmacytoid DCs (pDCs), are antigen-presenting cells that prime and regulate CTL responses. Anti-viral immune responses rely heavily on pDC-derived type I interferons (IFN) [88]; however in tumors pDCs often play potent immunosuppressive roles [89]. Tumor-infiltrating cDC increase T cell activation in lung cancer and melanoma patients, forming tertiary lymphoid clusters, and are associated with better outcomes [90–92]. Tertiary lymphoid clusters correlated with improved survival in pancreatic cancer [93]. CD103 (integrin αE)^+^ cDCs (Batf3-cDC, cDC1) are associated with CTL activation and increased overall survival for patients with breast, head and neck or lung cancer [94]. β-catenin signaling prevents tumor infiltration by DC and CTLs and imparts resistance to ICIs in melanoma [95]. In lung adenocarcinoma murine models, immunogenic chemotherapy (oxaliplatin-cyclophosphamide) has been reported to up-regulate toll-like receptor 4 (TLR-4) on tumor-infiltrating Batf3-cDCs, which leads to recruitment of CTLs and sensitization to ICIs [96].

#### Gut microbiota

Emerging evidence has suggested that the cross-talk between gut microbiota and immune cells plays a role in determining responses to ICI therapy [97]. The composition of the gut microbiome has been associated with response to ICI in pre-clinical models [98, 99]. In melanoma murine models, commensal Bifidobacterium has been reported to promote the efficacy of anti–PD-L1 therapy by augmenting the function of DCs leading to CTL priming and infiltration [98]. Recent studies in melanoma, lung and kidney cancer patients have demonstrated association of commensal gut microbiome with response to ICI and fecal transplant from responding patients in germ-free mice resulted in enhanced anti-tumor response [100–103]. In melanoma patients responding to ICI, more abundant species included Bifidobacterium, Collinsella, Enterococcus, Clostridiales, Rominococcus and Faecalibacterium while low levels of Akkermansia muciniphila were observed in epithelial cancers not responding to ICI [100–102]. Patients with a favorable gut microbiota had increased expression of cytolytic T cell markers, antigen processing and presentation, and increased ratio of CD8^+^ CTLs to FoxP3^+^CD4^+^ Tregs [104]. Modulation of gut microbiome can augment anti-tumor immunotherapy; however, there are several challenges including optimal composition of gut microbiome and therapeutic strategy to achieve that composition.

### Matrix remodeling and the inflamed immune microenvironment: untapped predictive and therapeutic potential

**Fig. 1:**
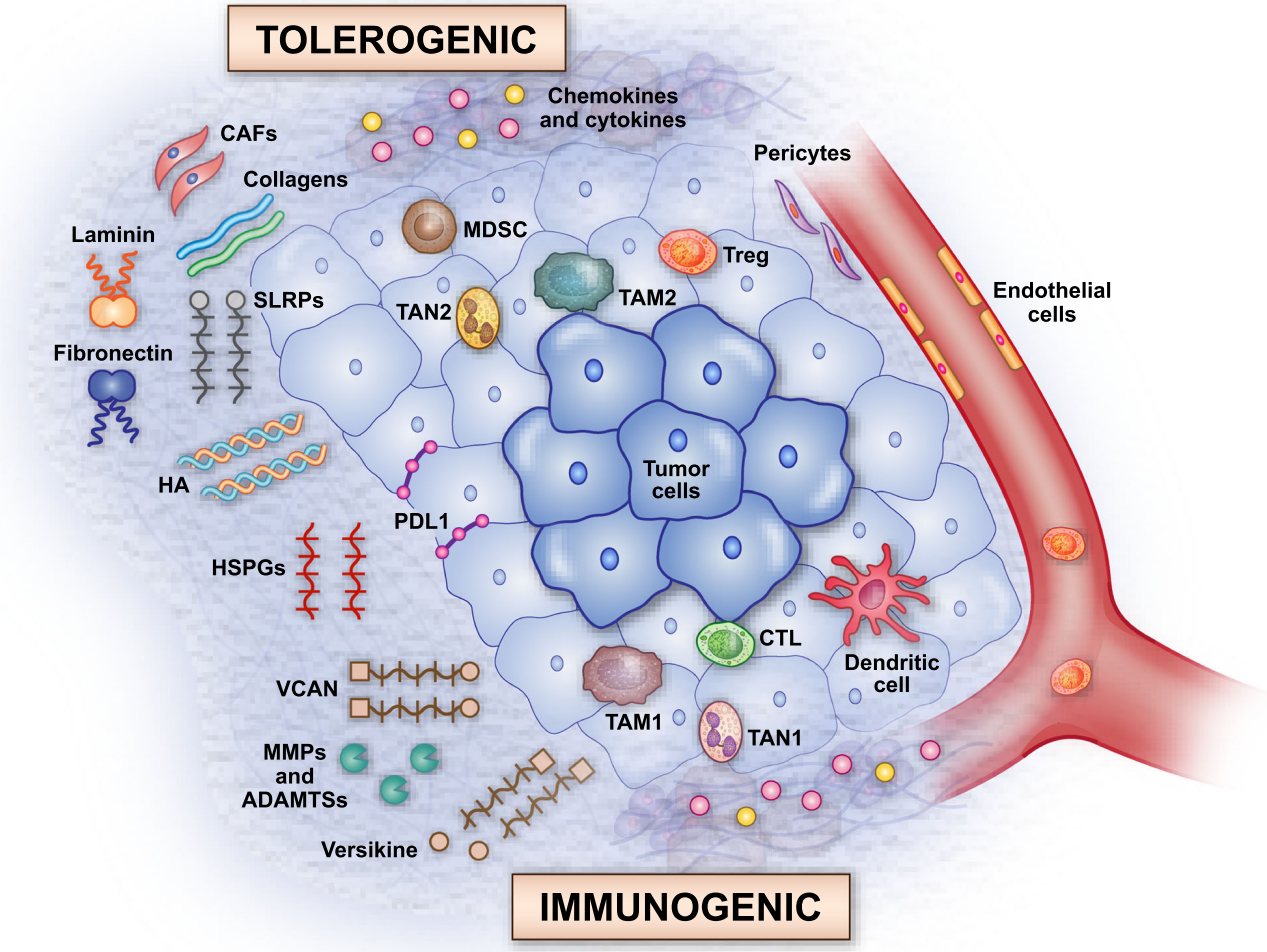
Extracellular matrix (ECM) and the inflamed tumor microenvironment. The TME is an intricate milieu of cells hosting the tumor, including infiltrating myeloid and lymphoid cells, stromal and mesenchymal cells, and ECM components. Matrix remodeling shapes the inflamed immune microenvironment. Tumor-infiltrating Tregs and regulatory myeloid cells, including MDSCs, TAMs and TANs, promote a tolerogenic TME. Tumor-infiltrating CTLs, dendritic cells, matrix components (like CAFs, HA, HSPGs, SLRPs, and VCAN), matrikines (e.g., versikine) and matrix-remodeling enzymes (MMPs and ADAMTSs) play a vital role in the generation and amplification of the host immune response. Abbreviations: TME; tumor microenvironment, ECM; extracellular matrix, CTL; cytotoxic lymphocytes, Treg; regulatory T cells, TAM 1 and 2; tumor-associated macrophages types 1 and 2, TAN 1 and 2; tumor-associated neutrophils types 1 and 2, MDSC; myeloid-derived-suppressor cells, PDL1; programmed cell death protein ligand 1, CAFs; cancer-associated fibroblasts, SLRPs; small leucine-rich proteoglycans, HA; hyaluronan, HSPGs; heparin sulfate proteoglycans, VCAN; versican, MMPs; matrix metalloproteinases, ADAMTSs, A disintegrin and metalloproteinase with thrombospondin motifs

The tumor microenvironment (TME) is an intricate milieu of cells hosting the tumor, including endothelial, mesenchymal and immune cells, along with the extracellular matrix (ECM) [105]. Both cellular and extracellular components of the TME play a pivotal role in tumor growth and metastasis [60], and matrix remodeling has an established role in tumor progression and invasion [106, 107]. Profiling of evolving TME in the ovarian cancer metastases revealed a distinct ECM-associated molecular signature comprising of 22 matrisome genes that predicted poor overall survival in 13 solid tumors suggesting a common and potentially targetable matrix response that influences the course of disease [108]. However, the contribution of the ECM remodeling in shaping the inflammatory and immune milieu of the tumor is only beginning to be systematically explored. (Fig. 1)

### Immune-cell trafficking in the TME: mechanisms and impact on immunotherapy responses

The trafficking of CTLs, Tregs and immune-suppressive myeloid cells is dependent on several factors encountered in the ECM, including matrix components, vascular endothelial cells and cell surface glycoproteins [105, 109]. Leakiness of tumor blood vessels regulated by endothelial cells and pericytes is important for cellular migration, including tumor-infiltrating immune cells [110]. Endothelial cells of the blood and lymphatic vessels proliferate in response to vascular endothelial growth factor (VEGF) resulting in neoangiogenesis [111, 112]. Angiogenic growth factors, including VEGF, decrease the expression of cell surface glycoproteins, including selectins, and intercellular and vascular cell adhesion molecules (ICAM-1 & 2 and VCAM-1) that mediate cell-cell surface interactions critical for CTL infiltration [110, 113]. Although endothelial cells impair CTL infiltration, they selectively promote transmigration of Tregs by upregulation of specific adhesion molecules and receptors like common lymphatic endothelial and vascular endothelial receptor-1 (CLEVER-1) [114, 115]. In renal cell carcinoma, inhibition of VEGF has resulted in improved survival through decrease of tumor-infiltrating Tregs and MDSCs [116, 117]. In renal cancer, combined PD-1 and VEGF blockade resulted in a response rate of 73%, almost double the response rates seen with ICI monotherapy [118]. VEGF inhibits T-cell development in thymus and VEGF blockade induces preferential commitment of lymphoid progenitors to the T lineage [119]. VEGF-A is proangiogenic but also plays a key role in immune modulation. VEGF-A enhances PD-1 expression on VEGFR-expressing CD8^+^ T cells, and promotes an immunosuppressive TME by inhibition of DC maturation and induction of Tregs and MDSCs [120]. VEGF-A blockade inhibits Treg proliferation in colorectal cancer [121]. In melanoma murine models, a combination of PD-1 with VEGF-A blockade induced a strong and synergic anti-tumor effect in tumors expressing high levels of VEGF-A [120]. Decreased VEGF-A gene expression has been observed in melanoma patients responding to ICI [40].

ECM cytoskeleton remodeling, structural plasticity and mechanical forces are increasingly recognized as crucial factors in immune cell trafficking, activation and immunological synapse formation [122]. Density of ECM and basement membrane composition is regulated by stromal matrix components and plays a key role in immune cell migration and spatial distribution [123, 124]. DCs and T cells are able to migrate along collagen type 1 fibrils independent of integrins and adhesion molecules whereas tumor and mesenchymal cells use protease and integrin-dependent migration as they are not able to penetrate dense fibrils [125–127]. In lung cancer, chemokine-dependent T cell infiltration occurs in loose fibronectin and collagen regions whereas it is impaired in dense matrix fibers surrounding tumor islets, leading to preferential stromal T cell accumulation and restricted tumor infiltration [128]. Hyaluronan interacts with T cells to facilitate adhesion and migration and this interaction is prevented by versican, highlighting interplay of stromal ECM components in leukocyte trafficking [129].

### Stromal and matrix-producer cells in the TME: immunomodulatory roles

Matrix components in the TME are produced by mesenchymal stem cells (MSCs), pericytes and cancer-associated fibroblasts (CAFs)***.*** Tumor-associated MSCs promote tumor growth and differentiate into pericytes and CAFs in response to stromal growth factors, including platelet-derived growth factor-β (PDGF-β) and fibroblast growth factors (FGF) [130, 131]. Pericytes promote structural dysfunction of blood vessels and suppress host immune response. In melanoma and colon cancer, pericytes promote T cell anergy [132]. In hepatocellular carcinoma, pericytes upregulate angiogenesis and facilitate the influx of immune suppressive cells [133]. In glioma, increase in pericytes results in decreased CTLs [134]. In melanoma, reduction in pericytes results in tumor infiltration of CTLs [135].

CAFs regulate the stromal matrix and serve as a primary source of matrix-associated proteins [131, 136]. CAFs express chemokines of CXC and CC family and cytokines of IL, IFN and TGF-β family. These orchestrate the immune-cell crosstalk and play an essential role in the infiltration of leukocytes in TME [105]. In gastric and colon cancer models, fibroblast activation protein-α (FAP)^+^ CAFs correlate with an immune suppressive phenotype, with increased CCL2 expression and decreased IFN-gamma and granzyme-B expression, promoting resistance to ICI therapy that is reversed by FAP^+^ CAF inhibition [137, 138]. However, in pancreatic cancer models, inhibition of CAFs resulted in immune suppression through infiltration of Tregs and increased tumor metastasis through disruption of the stromal fabric [139, 140]. Matrix stiffness by dense deposition of CAFs and shear stress has shown to activate the TGF-β pathway [141]. TGF-β in turn, modulates fibroblasts, collagens, and matrix enzymes to exert pleiotropic functional effects by either dampening or promoting T cell responses [131, 142, 143]. TGF-β also promotes metastasis by driving epithelial-to-mesenchymal transition [144].

### Extracellular matrix components and their role in tumor inflammation and tumor innate sensing

The extracellular matrix consists of hundreds of different components that together constitute the matrisome, including collagens, glycoproteins, and proteoglycans [145]. About one-third of matrisome proteins are tissue-specific both in normal and tumor extracellular matrix [146].

#### Collagens

Collagens provide tensile strength to the stroma and basement membrane. Collagen deposition is primarily mediated by fibroblasts and has a critical role in tumorigenesis and immune modulation. In colorectal cancer, tumor invasion and growth by increased collagen deposition and cross-linking has been observed [147]. Collagens act as functional ligands for the immune inhibitory receptor, Leukocyte Associated Ig-like Receptor-1 (LAIR-1), and tumor-expressed collagens can trigger immune inhibitory signaling via LAIR-1 [148].

#### Glycoproteins

There are several matrisome glycoproteins that mediate cellular interactions and define the structure of a tissue along with collagens. Laminins form the basement membrane that is a potentially important barrier to infiltration of immune cells in the matrix. Laminins, especially laminin 411 (α4) and 511 (α5), modulate migration and polarization of the leukocytes [149]. A higher ratio of laminin-α4 to laminin-α5 was seen in immune-tolerant lymph nodes and reducing laminin-α4 induced immune-mediated rejection in organ transplant murine models [150]. Laminin-α5 have been shown to inhibit leukocyte transmigration [151]. Laminins, in particular laminin 511, regulate structural intregrity of basement membrane and promote epithelial-to-mesenchymal transition (EMT) resulting in tumor invasion and metastases [152, 153]. Fibronectin and elastin comprise the interstitial matrix and are modulated by fibroblasts. Fibronectin is upregulated by angiogenic growth factors including VEGF. In lung cancer and melanoma pre-metastatic niches, hematopoietic cells bind with fibronectin via an integrin, VLA-4 (Very Late Antigen-4, CD49d/CD29), to form cellular clusters that precede the arrival of tumor cells, providing a permissive microenvironment for tumor growth [154].

#### Glucosaminoglycans

Glycosaminoglycans, including hyaluronan (HA), heparin, heparan sulfate, and chondroitin sulfate, are key macromolecules that affect cell migration and growth by acting directly on cell receptors or via interactions with growth factors [155]. HA is an abundant component of the matrix that modulates immune cells, by interactions with TLRs and CD44, and influences tumor growth via regulation of cellular differentiation and angiogenesis [156]. HA give dense architecture to TME impeding the infiltration of drugs and effector immune cells [157]. Functions of HA vary according to the size. Low molecular weight HA induces inflammation and angiogenesis, inhibits fibroblast differentiation and stimulates pattern-recognition receptors [156, 158–160]. High molecular weight HA is anti-angiogenic, promotes structural integrity, and suppresses the immune system by increasing activity of Tregs [156, 160, 161].

#### Proteoglycans

Proteoglycans contain repeating glycosaminoglycans that bind several cytokines and growth factors in the matrix. Heparan sulfate proteoglycans (HSPGs), including transmembrane (syndecan), glycosylphosphatidylinisotol (GPI)-anchored (glypican), secretory granule-derived (serglycin) and secreted HSPGs (perlecan, agrin and betaglycan), are large heterogeneous molecules that interact with growth factors, chemokines and structural proteins of the ECM to influence cellular differentiation and tumor progression [162–164]. Enzymatic degradation of HSPGs has been demonstrated to promote tumor infiltration and antitumor activity of chimeric antigen receptor (CAR)-T cells [165]. Small leucine-rich proteoglycans (SLRPs), include decorin, biglycan, fibromodulin, podocan, keratocan, and others. SLRPs can bind collagens and other matrix components; modulate immune cells by TLR, tumor necrosis factor-alpha (TNFα), and TGF-β pathways; and influence tumor growth and matrix remodeling by interaction with growth factors to modulate cellular differentiation and proliferation [166].

Versican (VCAN), a chondroitin sulfate proteoglycan, is normally present in small quantities in soft tissues but it accumulates in the inflamed cancerous and non-cancerous tissues [167]. It interacts with cells and stromal matrix components to regulate cell proliferation, migration, and activation. VCAN accumulation induces inflammation, and recruits and activates immune-suppressive myeloid cells [168–173]. It exerts tolerogenic effects by binding to TLR-2 in the tumor-infiltrating myeloid cells to promote immune evasion and tumor progression [26, 168, 174–177]. Increased stromal VCAN is associated with a decrease in tumor-infiltrating CTLs [178]. VCAN along with HA promotes neoangiogenesis in breast cancer [179]. High VCAN expression results in enhanced tumor invasion in gastric and cervical cancers [178, 180].

### Matrikines and matrix-remodeling enzymes: emerging players in anti-tumor immunity and immunotherapy

Cleavage of matrisome proteins by matrix-remodeling enzymes generates a wide variety of bioactive peptide fragments, the matrikines, which may act as chemokines or cytokines. Matrix metalloproteinases (MMPs) and adamalysins, including A disintegrin and metalloproteinases (ADAM) and A disintegrin and metalloproteinase with thrombospondin motifs (ADAMTS), are major families of the matrix enzymes that produce matrikines, many of which have unknown functions [106, 181].

Matrikines have a critical role in the infiltration of immune cells in TME and interact with immune regulators like TLRs. Elastin-derived matrikines act as chemokines for fibroblasts and up-regulate collagenase in lung cancer cells [182]. Collagen-derived fragments act as chemokines for immune cells and regulate the production of interleukins, in particular, IL-1β [183]. Laminin fragments influence EMT [184]. HA fragments promote inflammation in the TME by signaling through TLRs [160]. VCAN proteolysis, primarily by stromal cell-derived ADAMTS1, generates bioactive fragments, including versikine. It was shown to exert immune modulatory effects, by expression of inflammatory cytokines, IL1β and IL6, and T-cell chemoattractant, CCL2, in the myeloma niche [185]. VCAN proteolysis is associated with CTL infiltration in colorectal cancer, regardless of mismatch repair status, and versikine promotes T cell infiltration through regulation of Batf3-DCs [186].

MMPs have been associated with tumor progression and angiogenesis [187]. MMP-2 has been shown to promote tolerogenic polarization of DC through binding of TLR2 [188]. There have been several negative phase III clinical trials of MMP inhibitors, primarily because of non-specificity of drugs and complex context-specific roles of MMPs [189, 190]. ADAMTS genes have been found overexpressed, mutated or epigenetically silenced in several tumor types with varying degree of proteomic expression [191]. ADAMTS-mutated cases have higher chemotherapy response rates and better survival in ovarian cancer [192]. A lower ADAMTS13 gene expression has been associated with poor overall survival in bladder cancer patients who expressed a high level of PD-L1 [193]. Tissue inhibitors of metalloproteinases (TIMPs) antagonize matrix proteases and affect major signaling pathways by regulating proteolytic processing [194]. TIMP1 overexpression or TIMP3 silencing is consistently associated with cancer progression and poor prognosis [195]. Matrix proteases are also regulated by various transcriptional factors, cytokines and growth factors that orchestrate the cellular cross-talk and modulate immune and inflammatory responses [196].

### Multiple roles of the ECM in modulating the cancer-immunity cycle

**Fig. 2:**
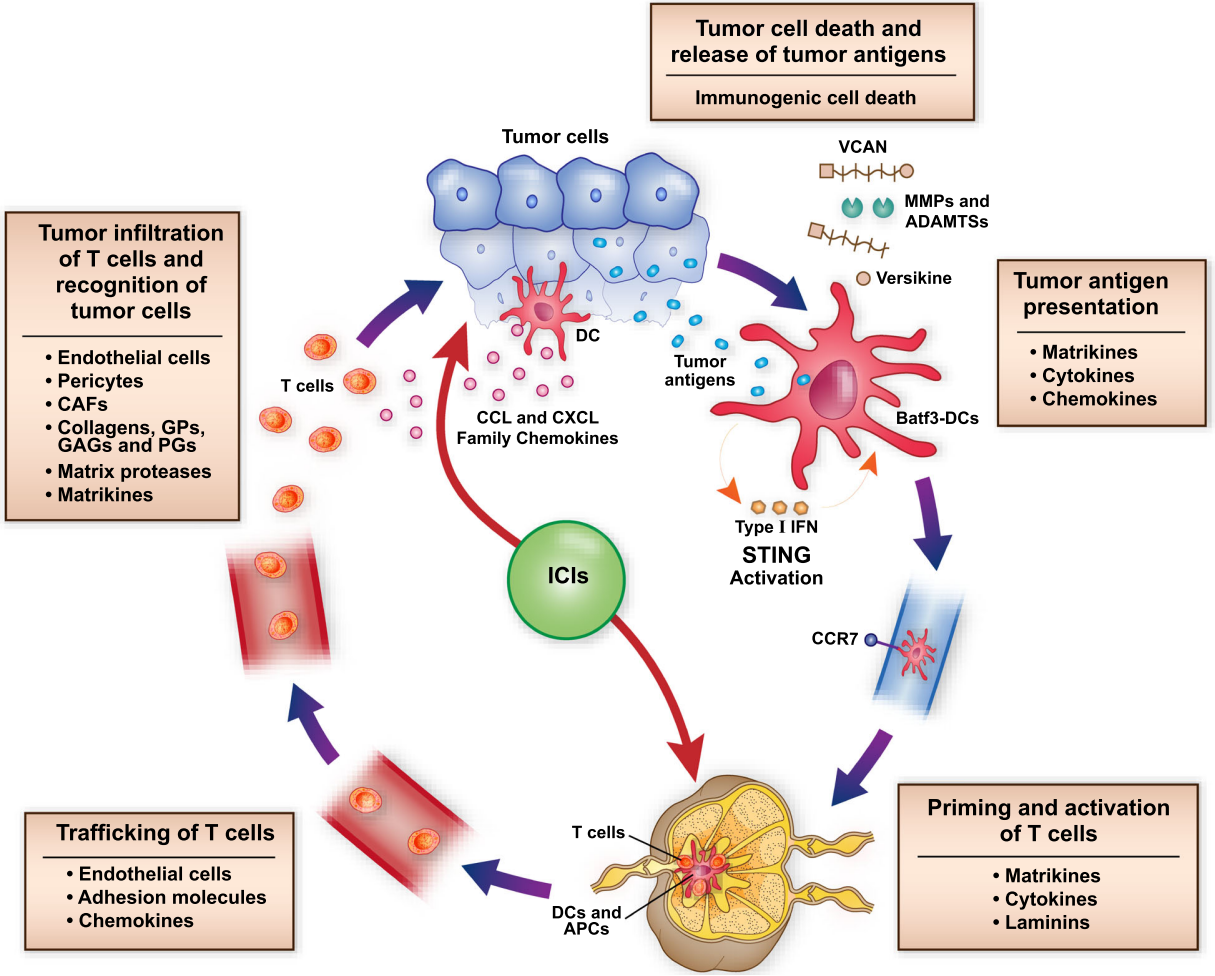
Multiple roles of the extracellular matrix (ECM) in modulating the cancer-immunity cycle. The cancer-immunity cycle progresses through tumor cell death and release of tumor antigens, tumor antigen presentation, priming and activation of T cells, trafficking of T cells, T-cell infiltration of tumor and recognition of tumor cells by effectors. Matrix remodeling shapes the inflamed immune microenvironment and plays a vital role at each step of the cancer-immunity cycle. Tumor antigen presentation and generation of the adaptive immune response depends on tumor-infiltrating Batf3-cDCs, matrikines, cytokines and chemokines of CCL and CXCL family. Versikine promotes differentiation of the potent immune-stimulator Batf3-cDCs. STING pathway activation, elicited by tumor cell-derived DNA, results in type I interferon (IFN) production and DC maturation. Endothelial cells, adhesion molecules and chemokines modulate trafficking of leukocytes. Stromal cells, including endothelial cells, pericytes and CAFs, and extracellular matrix components including collagens, GPs, GAGs and PGs, regulate infiltration and polarization of immune cells. Matrikines, cytokines and laminins regulate priming and activation of T cells. Matrix proteases and matrikines, including versikine, exert direct effects on immune cell polarization and activation. ICIs block immune checkpoints to induce anti-tumor immunity; however, the tumor matrix regulates generation and proliferation of the sustained host immune response. Matrix-derived immune biomarkers promise an innovative approach to predict response to novel immunotherapies. Abbreviations: Batf3-cDCs; Batf3-dependent classical dendritic cells, IRF8; interferon regulatory factor 8, IFN; interferon, STING; stimulator of interferon genes, CAFs; cancer-associated fibroblasts, GPs; glycoproteins, GAGs; glycosaminoglycans, PGs; proteoglycans, VCAN; versican, DCs; dendritic cells, APCs; antigen presenting cells

The cancer-immunity cycle, proposed by Chen and Mellman, provides a critical framework to evaluate anti-tumor immune response. It progresses through the immune-mediated tumor cell death and release of tumor antigens, tumor antigen uptake and presentation, priming and activation of T cells, trafficking of T cells, tumor infiltration of T cells and recognition of tumor cells [197]. The sustained immune response depends on the accumulation of immune-stimulatory factors and depletion of inhibitory factors. Matrix remodeling plays a vital role in cancer-immunity cycle by modulating immune regulatory feedback mechanisms. Stromal matrix components alter the immune milieu by several mechanisms and modulate differentiation, migration, infiltration and polarization of immune cells in the TME (Fig. 2).

Matrix-remodeling enzymes and matrikines, including versikine, exert direct effects on immune cell polarization and activation. They interact with immune receptors like TLRs and act as cytokines and chemokines to shape the direction and amplitude of the immune response. Recognition of tumor antigens and priming of CTLs is affected by matrikines via interactions with TLRs and DC function. Versikine appears to have a role in promoting local differentiation of the potent immunostimulator Batf3-cDC subset through IRF8 modulation [185, 186]. Endothelial cells, pericytes and adhesion molecules modulate trafficking and infiltration of leukocytes. CAFs secrete stromal elements, chemokines and cytokines, and activate TGF-β pathway that affects the recruitment and activation of myeloid-infiltrating cells. Collagens and glycoproteins, like laminin and fibronectin, regulate transmigration and polarization of immune cells in both lymphoid tissue and TME. Glycosaminoglycans, like HA, and proteoglycans, like HSPGs, SLRPs, and VCAN, support the development of an inflamed TME by diverse mechanisms that regulate activity of Tregs and immune-suppressive myeloid cells. A systematic understanding of matrix remodeling and the inflamed TME generated by stromal elements will help to identify investigational targets for development of novel immune biomarkers and combination immunotherapy.

### The case for matrix-based biomarkers: VCAN proteolysis to predict response to immune-modulating therapy

The detection of VCAN proteolysis in the TME may provide a convenient and reliable immune biomarker that can be utilized across tumor types. Its robust association with “T-cell inflammation” and likely, Batf3-cDC intratumoral density promises to select those patients most likely to respond to ICI and other immune-modulating therapies [186]. The regulated proteolysis of VCAN by ADAMTS-metalloproteinases exposes neoepitopes at the cleavage site that can be detected through neoepitope-specific antibodies [185]. Several attractive attributes underscore a potential role for VCAN proteolysis detection in this regard. Firstly, the proteolytic events do not appear to be tumor-specific and may be truly tumor-agnostic [185, 186]. Secondly, simple immunohistochemistry on standard, paraffin-embedded tissue is used, thus broadening the range of accessible samples to standard diagnostic samples collected in a variety of health care facility settings. Even decalcified tissue (e.g., myeloma bone marrow biopsies that resist attempts at detection of acid-sensitive epitopes such as PD-L1) can be robustly analyzed. Thirdly, the association between VCAN proteolysis and T-cell inflammation appears to be independent of neoantigen load- VCAN proteolysis predicted T-cell inflammation in both MSI and MSS colorectal cancers [186].

## Conclusions

The development of novel immunotherapies, including ICIs, was the twenty-first century breakthrough in oncology. Six ICI drugs have been FDA approved and many are in the pipeline. Although there have been durable remissions with the use of ICIs, less than third of the patients derive a benefit from these therapies. An often overlooked facet of immune regulation is the tumor matrix: a diverse and highly dynamic contributor that plays a vital role in the generation and proliferation of the host immune response. Exploring transcriptional imprint and proteomic expression of stromal matrix components may identify promising predictive and prognostic biomarkers. VCAN proteolysis is one emerging paradigm of matrix remodeling and immune modulation. Matrix-derived immune biomarkers promise to generate novel approaches to improve patient stratification and optimize therapeutic strategies employing novel immunotherapies.

## Abbreviations

ADAM: Adamalysins, including A disintegrin and metalloproteinases
ADAMTS: A disintegrin and metalloproteinase with thrombospondin motifs
CAR: Chimeric antigen receptor
CLEVER-1: Common lymphatic endothelial and vascular endothelial receptor 1
CTL: Cytotoxic lymphocytes (CD8^+^)
CTLA-4: Cytotoxic T lymphocyte antigen 4
CAFs: Cancer-associated fibroblasts
cDCs: Classical dendritic cells
DCs: Dendritic cells
ECM: Extracellular matrix
EMT: Epithelial-to-mesenchymal transition
FAP: Fibroblast activation protein
FGF: Fibroblast growth factors
FcγRs: Fc-gamma receptors
FDA: Food and Drug Administration
FOXP3: Forkhead-box P3
GPI: Glycosylphosphatidylinisotol
HA: Hyaluronan
HSPGs: Heparin sulfate proteoglycans
ICI: Immune checkpoint inhibitor
ICAM: Intercellular cell adhesion molecules
IFN: Interferons
IL: Interleukins
IL2Rα: Interleukin-2 receptor chair-alpha
LAIR-1: Leukocyte Associated Ig-like Receptor-1
MMPs: Matrix metalloproteinases
MSCs: Mesenchymal stem cells
mAb: Monoclonal antibodies
MSI: Microsatellite instability
MSS: Microsatellite-stable
MDSCs: Myeloid-derived-suppressor cells
NSCLC: Non-small cell lung cancer
pDCs: Plasmacytoid dendritic cells
PDGF-β: Platelet-derived growth factor-β
PD-1: Programmed cell death protein 1
PD-L1: Programmed cell death protein ligand 1
PD-L2: Programmed cell death protein ligand 2
SLRPs: Small leucine-rich proteoglycans
Tregs: Regulatory T cells (CD4^+^)
TIMPs: Tissue inhibitors of metalloproteinases
TNFα: tumor necrosis factor-alpha
TCR: T cell receptor
TLR: Toll-like receptor
TGF-β: Transforming growth factor-beta
TME: Tumor microenvironment
TAMs: Tumor-associated macrophages
TANs: Tumor-associated neutrophils
VCAM: Vascular cell adhesion molecules
VCAN: Versican
VEGF: Vascular endothelial growth factor
VLA-4: Very Late Antigen-4
